# Severe Toxicities From Definitive Chemoradiotherapy for Oropharyngeal Cancer in a Patient With Comorbid Sjögren’s Syndrome

**DOI:** 10.7759/cureus.92966

**Published:** 2025-09-22

**Authors:** Mitsutoshi Ooishi, Osamu Togao, Koichi Hirakawa, Yoichiro Sugiyama

**Affiliations:** 1 Department of Radiology, Faculty of Medicine, Saga University, Nabeshima, JPN; 2 Department of Radiology, National Hospital Organization Ureshino Medical Center, Ureshino, JPN; 3 Department of Otolaryngology-Head and Neck Surgery, Faculty of Medicine, Saga University, Nabeshima, JPN

**Keywords:** collagen vascular disease, concurrent chemoradiation therapy, head and neck cancer (hnc), primary sjögren's syndrome, radiation-induced toxicity

## Abstract

Radiotherapy in patients with a collagen vascular disease (CVD) requires careful consideration due to the potential for exacerbated treatment-related toxicities. We report the case of a 43-year-old Japanese woman with human papillomavirus (HPV)-positive oropharyngeal cancer and a history of Sjögren's syndrome (SjS) who underwent definitive chemoradiotherapy with high-dose cisplatin (100 mg/m^2^ every three weeks) and three-dimensional conformal radiation therapy (3D-CRT) to a total dose of 70 Gy in 35 fractions. Beginning midway through treatment (around 30-40 Gy), she developed severe acute toxicities, including Grade 3 radiation dermatitis and confluent mucositis, per the Common Terminology Criteria for Adverse Events (CTCAE) v5.0. This was followed by significant late toxicities, including bilateral middle ear effusion associated with chronic otitis media and persistent xerostomia that continued to impact her oral intake. This patient's case highlights the potential for severe treatment-related toxicity in patients with SjS undergoing standard chemoradiotherapy for head and neck cancer, and it underscores the importance of careful pre-treatment evaluation and individualized planning.

## Introduction

Collagen vascular diseases (CVDs) are a heterogeneous group of systemic autoimmune disorders characterized by chronic inflammation, immune dysregulation, and progressive injury to multiple organ systems. The principal conditions include rheumatoid arthritis (RA), systemic lupus erythematosus (SLE), systemic sclerosis (SSc), Sjögren’s syndrome (SjS), and polymyositis/dermatomyositis (PM/DM). Among CVDs, RA is the most prevalent condition. According to the Global Burden of Disease Study 2021, the global age-standardized prevalence in 2020 was approximately 209 cases per 100,000 population, with a clear female predominance (female-to-male ratio of 2.5:1) [1].

SjS, once considered rare, is now recognized as one of the most common autoimmune diseases. Its prevalence is estimated to range from 0.1% to 4.8% in the United States [2], whereas nationwide surveys in Japan have reported a prevalence of approximately 0.05% [3]. The pathobiology of CVDs reflects a multifactorial interplay of genetic predisposition, autoantibody production, cytokine-driven inflammation, and environmental triggers [4,5]. Histopathologically, these disorders are characterized by persistent inflammation, vascular injury, and aberrant collagen deposition, ultimately leading to fibrosis and progressive organ dysfunction [6]. Management of CVDs generally relies on immunosuppressive and immunomodulatory therapies, including corticosteroids, conventional disease-modifying antirheumatic drugs, and, more recently, biologic agents, tailored according to disease activity and organ involvement [2,4].

Importantly, patients with CVDs appear to have an increased risk of malignancy. This risk is attributed to chronic autoimmune-mediated tissue damage, impaired clearance of oncogenic viruses, and the cumulative effects of long-term immunosuppressive therapy [5]. In SjS, patients appear to have an increased risk of malignancy, most notably a somewhat higher likelihood of developing non-Hodgkin B-cell lymphoma [5], while elevated risks of various solid tumors have also been reported [7].

Radiotherapy in patients with a CVD is clinically challenging, as multiple studies have suggested an increased risk of both acute and late toxicities in this population [8,9]. Shaikh et al. reported a significantly higher incidence of Grade ≥2 toxicities among patients with CVDs compared to controls, particularly in patients with RA, SLE, or SSc, especially when irradiated regions included the breast, pelvis, thorax, or skin [8]. However, reports of radiotherapy performed for patients with SjS remain rare, and the safety profile in this population is not well defined. We present a case of HPV-positive oropharyngeal cancer in a patient with SjS who developed severe radiation dermatitis, mucositis, and xerostomia during definitive chemoradiotherapy. This report aims to contribute to the limited evidence regarding the safety of radiotherapy in patients with SjS, particularly those undergoing definitive chemoradiotherapy for head and neck cancers.

## Case presentation

A 43-year-old Japanese woman with a history of SjS presented with a left neck mass. Approximately 20 years prior, she had been diagnosed with SjS based on symptoms of oral and ocular dryness, a positive Schirmer’s test, decreased salivary gland function on scintigraphy, and positive anti-SSA (Ro) and anti-SSB (La) antibodies. She had no major organ involvement but experienced oral dryness, arthralgia, and Raynaud's phenomenon. Over the six years prior to her presentation, recurrent finger swelling and joint pain were managed with low-dose prednisolone (PSL) (2.5-5 mg/day); at presentation, she was on PSL 5 mg/day. Three months before her presentation, the patient noted swelling of the left cervical lymph nodes.

**Figure 1:**
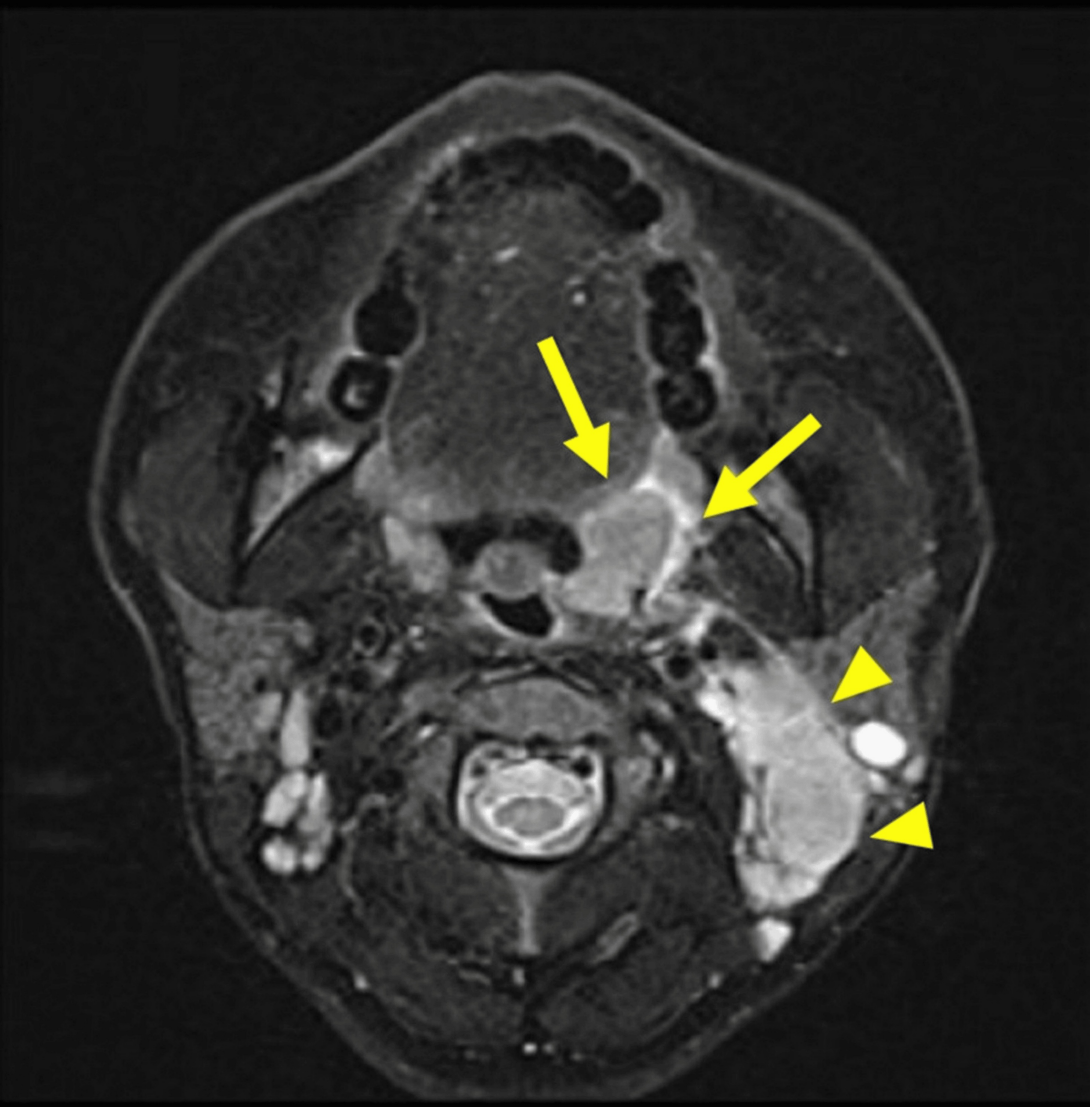
Axial fat-suppressed T2-weighted MRI image at presentation. A tumor measuring approximately 25 × 16 mm was identified in the left palatine tonsil (yellow arrow), along with multiple enlarged lymph nodes in the left cervical region, the largest measuring up to 5 cm (yellow arrowhead). The patient was diagnosed with left-sided oropharyngeal cancer originating from the palatine tonsil. The tumor was p16-positive and classified as clinical T2N1M0, Stage I, according to the TNM classification of Malignant Tumors, 8th edition [10].

An examination revealed enlargement of the left palatine tonsil and cervical lymphadenopathy. A biopsy of the patient's tonsil demonstrated p16-positive squamous cell carcinoma. Magnetic resonance imaging (MRI) showed a 25 × 16-mm tumor in the left tonsil and multiple enlarged cervical lymph nodes, the largest measuring 5 cm (Figure 1).

Positron emission tomography/computed tomography (PET/CT) with 18F-fluorodeoxyglucose (FDG) showed no distant metastasis. The laboratory findings at presentation are shown in Table 1. The following were observed while the patient was receiving PSL at 5 mg/day: elevated inflammatory markers, hypoalbuminemia suggesting malnutrition, and increased serum IgG levels.

**Table 1:** Laboratory data at presentation. WBC: white blood cell count; RBC: red blood cell count; Hb: hemoglobin; Plt: platelets; TP: total protein; Alb: albumin; AST: aspartate aminotransferase; ALT: alanine aminotransferase; BUN: blood urea nitrogen; Cr: creatinine; CRP: C-reactive protein; Ig: immunoglobulin.

Parameters	Value	Reference range
Complete blood count		
WBC (/μL)	5,400	3300-8600
RBC (×10⁶/μL)	4.09	3.86-4.92
Hb (g/dL)	12.0	11.6-14.8
Plt (×10³/μL)	310	158-348
Blood chemistry		
TP (g/dL)	9.0	6.6-8.1
Alb (g/dL)	3.1	4.1-5.1
AST (U/L)	29	13-30
ALT (U/L)	19	7-23
BUN (mg/dL)	10.8	8–20
Cr (mg/dL)	0.6	0.46-0.79
CRP (mg/dL)	4.27	0.00-0.14
Immunology		
IgG (mg/dL)	3,732.0	861.0-1747.0
IgA (mg/dL)	249.1	93.0-393.0
IgM (mg/dL)	118.3	50.0-269.0

We diagnosed HPV-positive oropharyngeal cancer, clinical T2N1M0, Stage I per the Union for International Cancer Control (UICC)'s TNM Classification of Malignant Tumours, 8th edition [10]. At this stage, both surgical and non-surgical approaches may be considered for HPV-positive oropharyngeal carcinoma. At our institution, surgical treatment was initially discussed by the otolaryngology department; however, reconstruction of the pharyngeal wall was deemed necessary, which would have resulted in a highly invasive procedure. In addition, due to limited surgical slots, the expected waiting period for surgery was several months. Given the patient’s rapid tumor progression, it was necessary to initiate treatment without delay. In fact, radiotherapy was started only two days after the planning CT was performed. Furthermore, while intensity-modulated radiation therapy (IMRT) is now the standard technique to reduce treatment-related toxicities, manpower shortages at our institution made it difficult to initiate IMRT planning immediately. Therefore, three-dimensional conformal radiation therapy (3D-CRT) was selected as the most feasible option to enable prompt treatment initiation.

**Figure 2:**
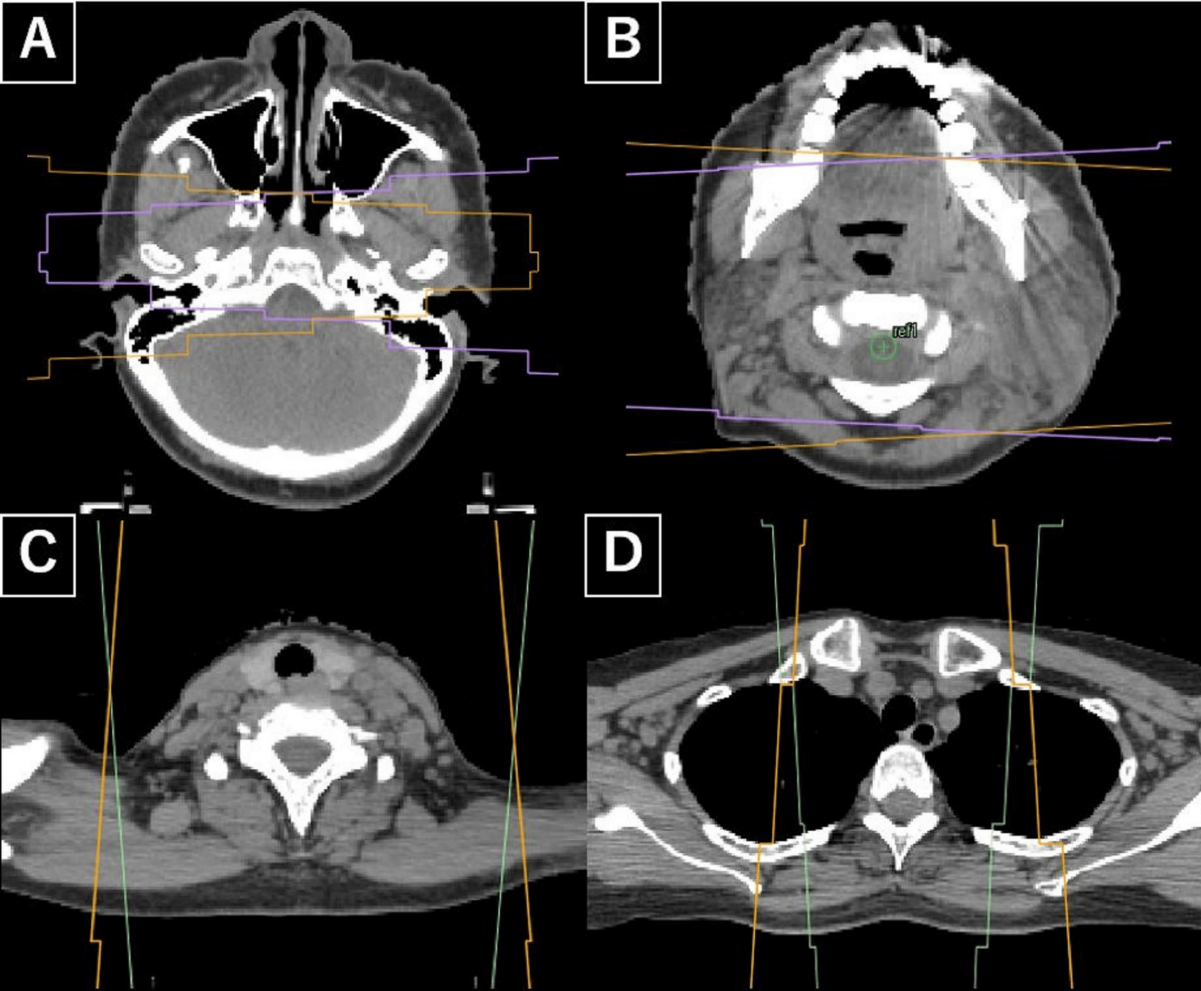
Radiation field borders (3D-CRT). The superior border was set at the skull base including the nasopharyngeal roof (A), the anterior border at the retromolar trigone, and the posterior border at the sternocleidomastoid muscle (B). At the lower cervical level, the field encompassed the spinal accessory nerve region, lower internal jugular region, and the supraclavicular fossa (C, D). The CTV included bilateral nodal levels Ib–IVa, the spinal accessory nerve region, the retropharyngeal nodes (given the risk of retropharyngeal involvement in oropharyngeal cancer), and the supraclavicular fossa, as contralateral nodal involvement could not be completely excluded due to several prominent but PET-negative nodes on the opposite side. The ipsilateral submandibular region (level Ib) was also included because of the bulky metastatic level II node with extranodal extension, and the supraclavicular fossa was covered to account for potential inferior nodal spread. Field borders are illustrated on the axial CT images. 3D-CRT: three-dimensional conformal radiation therapy; CTV: clinical target volume

Accordingly, definitive chemoradiotherapy was planned using high-dose cisplatin (100 mg/m^2^ every three weeks) and external beam radiotherapy to 70 Gy in 35 fractions. The whole neck received 40 Gy in 20 fractions using 3D-CRT, followed by a boost of 30 Gy in 15 fractions to the primary tumor and involved lymph nodes. The radiation field borders of the whole neck are shown in Figure 2.

The superior border was set at the skull base, including the roof of the nasopharynx, to encompass the nasopharyngeal mucosa in consideration of the potential superior extension of the primary tumor (Figure 2A). At the oropharyngeal level, the anterior border was placed at the retromolar trigone and the posterior border at the posterior edge of the sternocleidomastoid muscle (Figure 2B). At the lower cervical level, the lateral border extended to include the spinal accessory nerve region and the supraclavicular fossa (Figure 2C), and the inferior border encompassed the lower internal jugular region and the supraclavicular fossa (Figure 2D). These field borders were delineated on the axial CT images (Figure 2).

**Figure 3:**
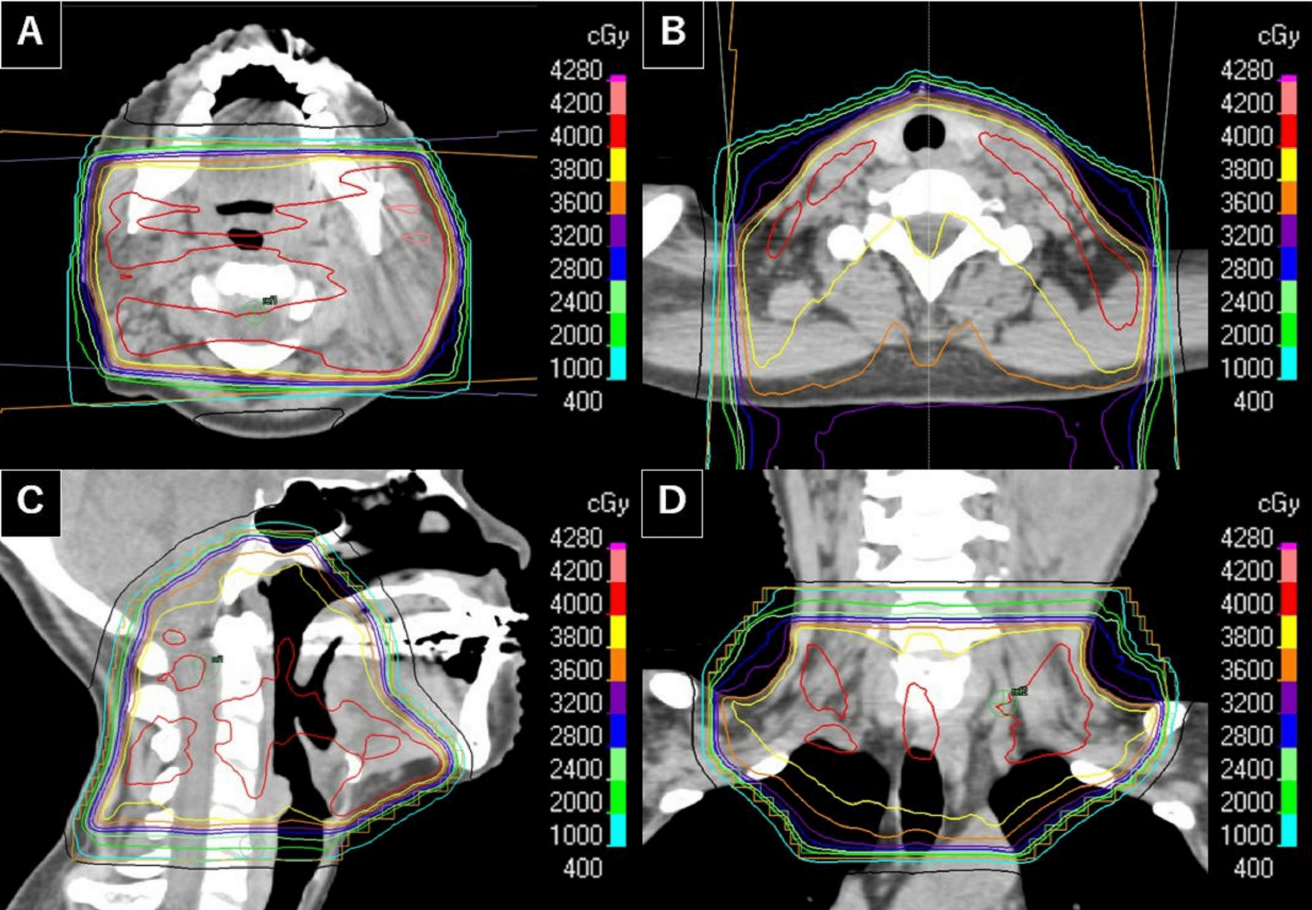
The dose distribution of whole-neck irradiation using the half-beam technique (A-D), delivered with 6 MV X-rays. Axial (A, B), sagittal (C), and coronal (D) CT images show the planned radiotherapy fields and dose gradients for comprehensive neck coverage. Isodose lines are indicated according to the dose scale (right panel). The 40 Gy line is depicted in red, the 28 Gy line in blue, and the 10 Gy line in light blue. The superior border of the irradiation field extended to the skull base including the nasopharynx; the anterior border was set anterior to the retromolar trigone and submandibular region; the posterior border covered the sternocleidomastoid muscle posteriorly including the spinal accessory nerve region; and the inferior border extended to the supraclavicular fossa.

A bulky metastatic lymph node was identified in the left level II region, with central necrosis and findings suggestive of extranodal extension; therefore, the left level Ib was included in the clinical target volume (CTV). In addition, nodal enlargement was observed along the course of the left spinal accessory nerve, so the left spinal accessory nerve region and the supraclavicular fossa were also covered. On the right side, several cervical lymph nodes appeared prominent but did not show FDG uptake on PET-CT; although the case was staged as cN1, the possibility of occult metastasis could not be completely excluded, which justified including the contralateral nodal levels. In addition, given the rapid progression of the tumor and the recognized risk of retropharyngeal involvement in oropharyngeal cancer, bilateral nodal levels Ib-IVa, the spinal accessory nerve region, the retropharyngeal nodes, and the supraclavicular fossa were included in the CTV. Dose distributions can be seen in Figure 3.

**Figure 4:**
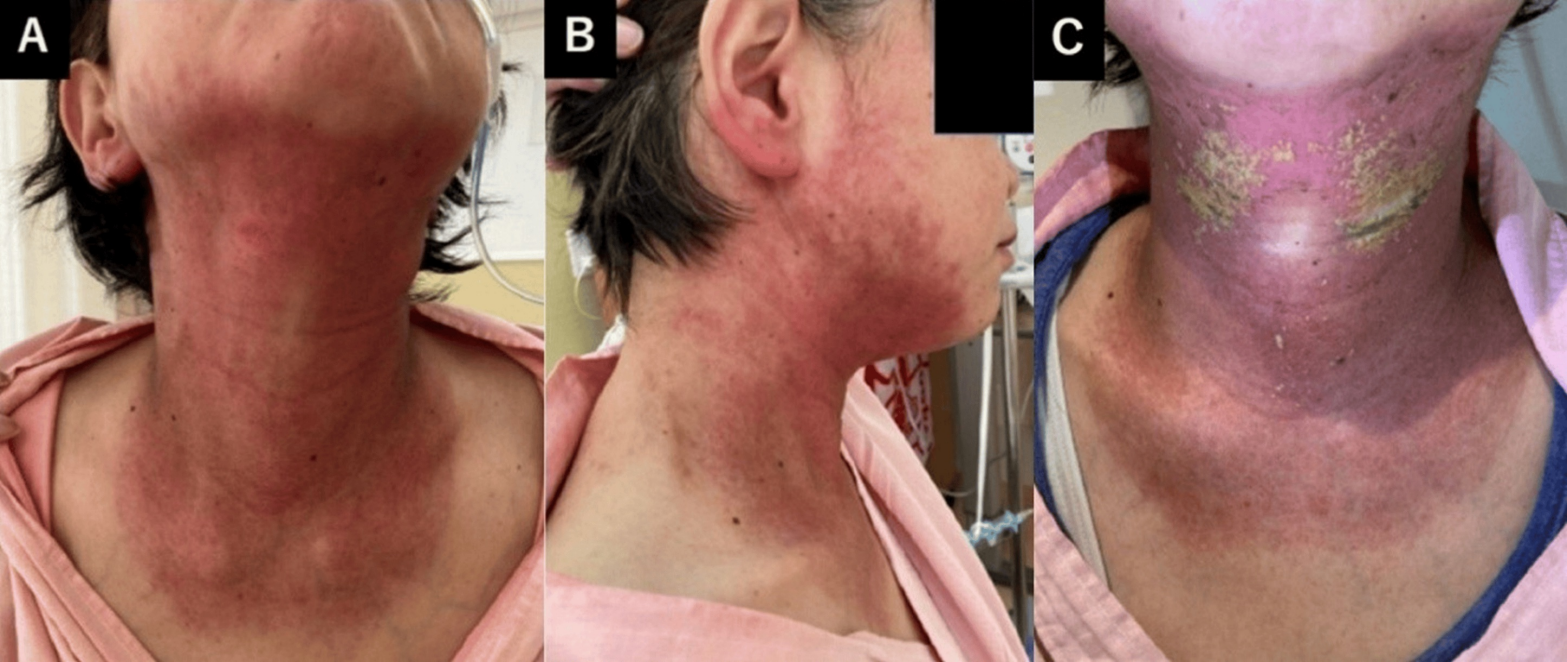
Neck skin findings during the patient's radiotherapy. Images at 40 Gy in 20 fractions (A, B) show marked skin erythema within the whole-neck irradiation field. Widespread erythema and partial skin erosion were observed in the cervical region, consistent with Grade 2 radiation dermatitis (CTCAE v5.0). By the timepoint at which 44 Gy in 22 fractions had been delivered (C), purulent contamination developed at the eroded cervical skin, and the dermatitis progressed to Grade 3.

The tumor began to shrink at an early point during this treatment. When 16 Gy had been reached, acetaminophen (1,500 mg/day) was started for the patient's glossitis. After 18 Gy, a mouthwash prepared by mixing glycerin, xylocaine, and distilled water was used to relieve mucositis-related pain. At 30 Gy, worsening odynophagia developed, managed with acetaminophen (3,000 mg/day) and tramadol (150 mg/day). Concurrent skin erythema appeared. At 36 Gy, the patient was referred to the palliative care department for the management of radiation-induced mucositis, and a fentanyl patch (1 mg) was initiated for mucositis-related pain. By 38 Gy, progressive dysphagia necessitated enteral nutrition due to Grade 3 mucositis (Common Terminology Criteria for Adverse Events (CTCAE) v5.0 [11]), and a consultation with the Nutrition Support Team (NST) was requested to optimize nutritional management. At 40 Gy, the patient's worsening pharyngeal pain required an increased fentanyl dose (2 mg), and Grade 2 radiation dermatitis developed, with widespread erythema and localized skin erosion in the cervical region (Figure 4A, 4B). Topical management included Melolin® dressings, azulene ointment, and betamethasone butyrate propionate ointment (Antebate®), applied twice daily. However, a purulent infection developed at the eroded skin site, progressing to Grade 3 dermatitis (Figure 4C).

**Figure 5:**
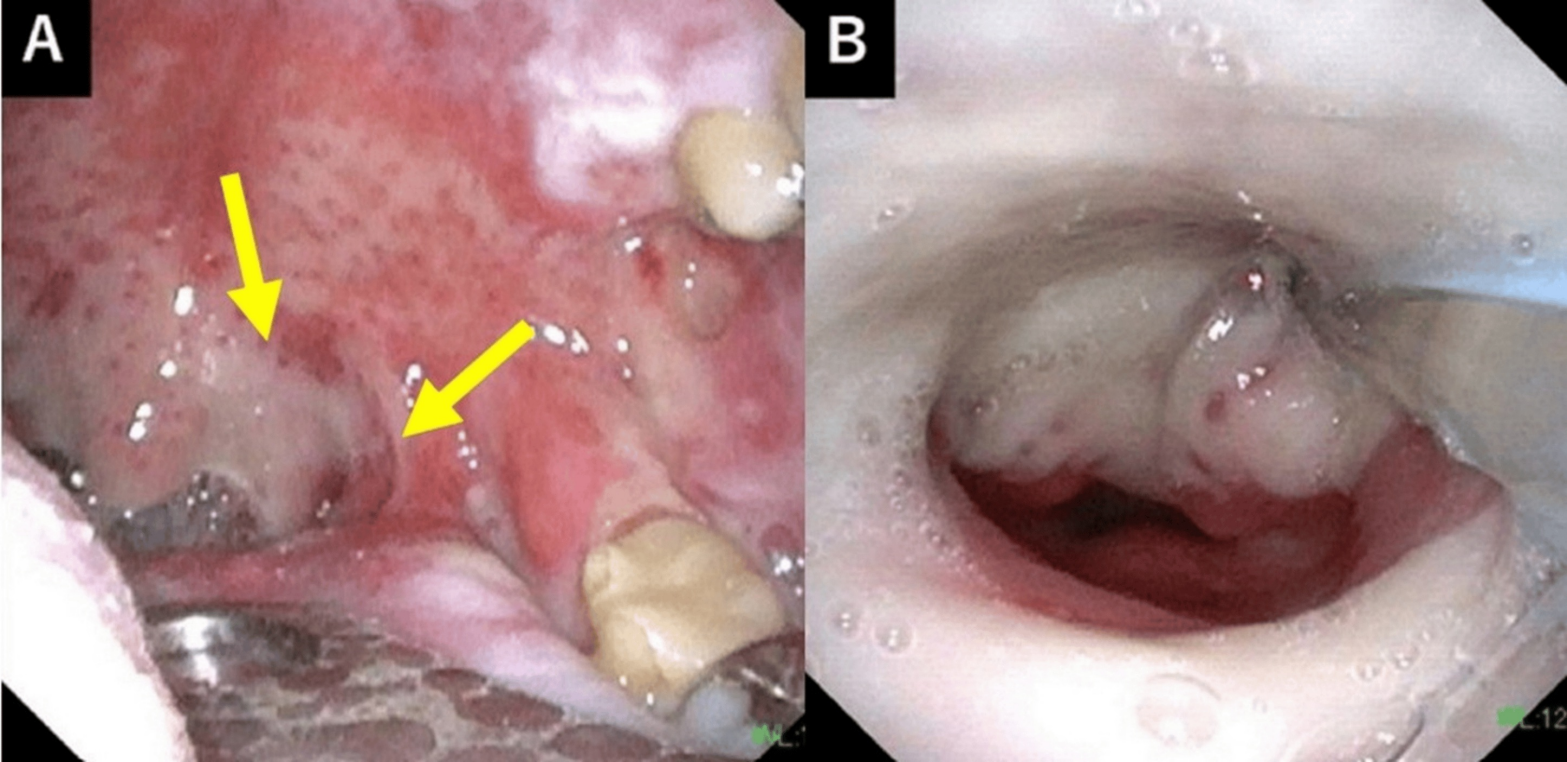
Endoscopic findings during radiotherapy. At 44 Gy in 22 fractions, a thick white coating was observed on the left palatine tonsil in the oral cavity (A, yellow arrow). At the laryngeal level (B), circumferential confluent mucositis consistent with Grade 3 radiation toxicity was noted, according to CTCAE v5.0.

An endoscopic examination at 44 Gy revealed Grade 3 mucositis in the oral cavity and the pharyngolaryngeal region (Figure 5).

**Figure 6:**
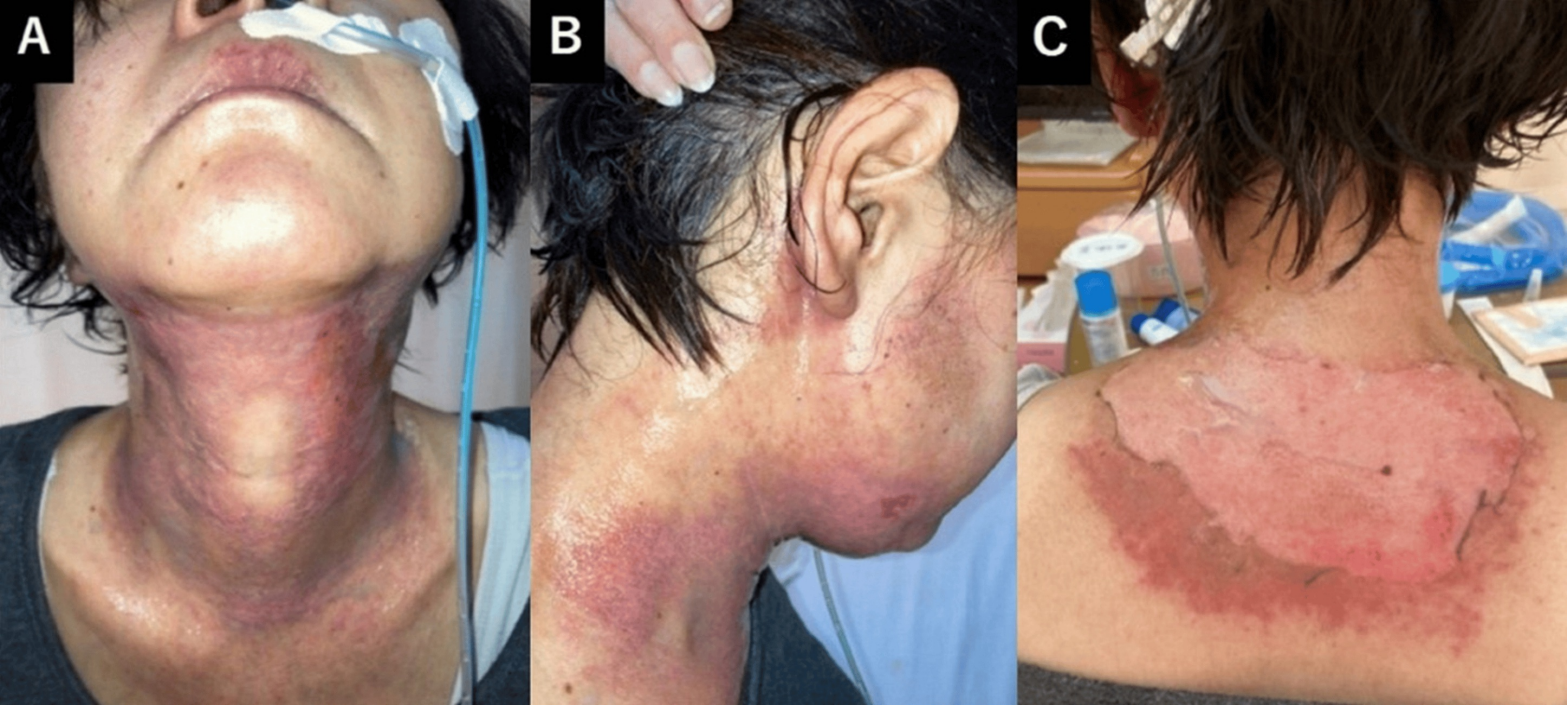
Neck skin findings at 54 Gy in 27 fractions. Extensive erythema and erosion persisted on the posterior neck (C), most of which was outside the irradiation field beyond 40 Gy, while partial re-epithelialization was observed in the anterior neck (A, B).

Although the treatment team proposed limiting the radiation field to reduce toxicity, the patient wished to proceed as planned. Mucositis persisted at Grade 3 until the treatment's completion. At 54Gy, epithelial erosion persisted in the posterior cervical skin, with partial re-epithelialization anteriorly (Figure 6). At the same time, a dermatology consultation was obtained, and the management of radiation dermatitis was reconfirmed to include the application of azulene ointment and betamethasone butyrate propionate ointment (Antebate®) twice daily, together with Melolin® dressings.

Despite significant toxicity, the patient completed radiotherapy with a reduced third cisplatin dose (80 mg/m^2^ due to myelosuppression). PSL (5 mg/day) was continued throughout the treatment for SjS management. Post-treatment PET/CT showed a complete response at the primary site and a partial response in cervical lymph nodes. Three months later, the patient underwent left neck dissection (levels I-V), which revealed no residual disease, confirming pathological N0 status. After the neck dissection, transient bilateral mandibular edema developed, which was suspected to be attributable to the surgical procedure itself. Apart from this, no obvious complications were observed, and the prior severe radiation-related toxicities did not appear to have any particular impact on the surgical procedure or postoperative course.

Late adverse effects included bilateral middle ear effusion associated with chronic otitis media, requiring myringotomy and tympanostomy tubes, resulting in the tympanic membrane perforations persisted (CTCAE v5.0 Grade 3). Persistent xerostomia interfered with the patient's oral intake (Grade 2), but her laryngeal function remained intact. Although the patient reported worsening oral dryness compared to before radiotherapy, no objective salivary function tests were performed before or after treatment. Mild cervical skin fibrosis was also observed (Grade 1). The patient has remained disease-free for approximately three years.

## Discussion

Radiotherapy in patients with a CVD must be administered with caution, as several reports have demonstrated a heightened risk of both acute and late toxicities associated with radiotherapy in this population [8,9,12]. Patients with active SLE or SSc in particular appear to be prone to increased late radiation-related toxicities [12], making radiotherapy a relative contraindication in some cases. Nevertheless, tumor control often remains a crucial determinant of the prognosis, and radiotherapy may be necessary despite its potential risks.

Several reports have described radiotherapy in patients with head and neck cancer and an underlying CVD. Lee et al. treated locally advanced oropharyngeal cancer in a patient with SLE who developed Grade 4 mucositis during chemoradiotherapy [9]. Conversely, Yamaguchi et al. described two cases of patients with head and neck cancer with dermatomyositis treated with chemoradiotherapy, with neither patient experiencing Grade ≥3 toxicities [13]. Huang et al. reported a significantly increased incidence of acute skin toxicity in 86 patients with nasopharyngeal carcinoma and underlying dermatomyositis or polymyositis compared to healthy controls [14]. However, reports of radiotherapy specifically in patients with SjS and head and neck cancer remain rare.

The radiotherapy with concurrent cisplatin for oropharyngeal cancer in our patient with SjS led to severe adverse events. These toxicities were unlikely to be the result solely of individual radiosensitivity, suggesting a potential contribution of the patient's underlying SjS. To further contextualize these findings, it is useful to compare the observed toxicities with those reported in large prospective trials, such as RTOG 1016 [15]. It should be noted that RTOG 1016 enrolled a standard patient population, and cases with collagen vascular diseases were unlikely to have been included. In the cisplatin arm, grade ≥3 oral mucositis, pharyngeal mucositis, and radiation dermatitis were reported in 41.5%, 13.6%, and 8.0%, respectively. In our case, grade 3 oral and pharyngeal mucositis developed in the latter half of treatment, comparable to, and possibly more pronounced than, those reported in RTOG 1016, whereas grade 3 dermatitis appeared more pronounced, suggesting that SjS may have exacerbated radiation-induced skin toxicity. Regarding late effects, our patient maintained swallowing function despite persistent xerostomia (cf. 4.4% severe dysphagia in RTOG 1016), and the hearing impairment was conductive due to bilateral middle ear effusion associated with chronic otitis media rather than cisplatin-related sensorineural loss (6.3% in RTOG 1016).

This interpretation of enhanced toxicity in SjS is further supported by several published reports (Table 2). For example, Hareyama et al. described a patient with nasopharyngeal carcinoma and SjS combined with mixed connective tissue disease (MCTD) who developed a retropharyngeal abscess and ulceration after treatment with cisplatin and 5-fluorouracil [16]. In another report, Hernandez et al. described Grade 3 dermatitis and pneumonitis in a patient with SjS and rheumatoid arthritis undergoing postoperative radiotherapy for breast cancer [17]. Severe oral dryness, mucositis, and dermatitis in a patient with nasopharyngeal carcinoma and comorbid SjS treated with chemoradiotherapy were reported by Lai et al. [18]. Furthermore, multiple cases of severe radiation pneumonitis and esophagitis in patients with SjS and MCTD receiving thoracic radiotherapy were described in a report by Diao et al. [19]. Although limited, these reports suggest that patients with SjS may be predisposed to enhanced radiation-related toxicities. 

**Table 2:** Cases of radiotherapy for patients with collagen vascular disease, cancer, and Sjögren's syndrome 5-FU: 5 fluorouracil, CDDP: cisplatin, CVD: collagen vascular disease, MCTD: mixed connective tissue disease, n.a.: not available, RA: rheumatoid arthritis, SjS: Sjögren's syndrome.

Authors, year	Age	Sex	CVD	Primary cancer	Chemotherapy	Prescribed radiation dose/fractionation	Adverse events
Hareyama et al., 1996 [16]	64	M	SjS/MCTD	Nasopharyngeal cancer	CDDP, 5-FU	66.6 Gy/37 fractions	Retropharyngeal abscess; ulceration of the mucosal membrane on the posterior wall of the oropharynx
Hernandez et al., 2006 [17]	53	F	SjS/RA	Breast cancer	Not administered	Whole breast: 45 Gy/25 fractions, Boost: 14 Gy/7 fractions	Grade 3 pneumonitis, dermatitis
Lai et al., 2014 [18]	58	F	SjS	Nasopharyngeal cancer	CDDP	70 Gy/35 fractions	Severe oral dryness, mucositis, dermatitis
Diao et al., 2017 [19]	n.a.	n.a.	SjS/MCTD	Lung cancer	n.a.	n.a.	≥ Grade 3 esophagitis (2 patients); ≥ Grade 3 pneumonitis (1 patient)
Present Case	43	F	SjS	Oropharyngeal cancer	CDDP	70 Gy/35 fractions	Grade 3 dermatitis, mucositis, otitis media; Grade 2 xerostomia

In line with these observations, our case demonstrated prominent acute toxicities such as severe mucositis and dermatitis, while late effects included exacerbation of xerostomia and chronic otitis media. Importantly, severe late skin fibrosis was not observed. By contrast, SLE is associated with severe acute reactions, such as mucositis and dermatitis, and may also lead to late sequelae, including soft tissue and skin fibrosis, as well as gastrointestinal complications such as bowel obstruction or perforation [8,9,12,20]. In SSc, late fibrotic and necrotic complications are particularly prominent, as confirmed in a recent case series of head and neck cancer patients [8,21]. Regarding RA, a recent meta-analysis demonstrated increased risks of late complications, including poorer cosmetic outcomes after breast irradiation [8]. Collectively, these comparisons suggest that in SjS, acute mucosal and cutaneous toxicities and late salivary gland dysfunction may be more apparent than fibrotic reactions, although the evidence remains limited. By contrast, SSc and SLE show more fibrotic profiles, and RA also carries elevated risks of late complications.

Beyond the general toxicity profile, a noteworthy finding in our case was the development of posterior cervical dermatitis outside the high-dose field. In our case, posterior cervical dermatitis worsened after 40 Gy despite being outside the high-dose field. This course suggests the possibility of radiation recall dermatitis (RRD), although exacerbation of radiation dermatitis due to underlying SjS must also be considered. RRD is a rare inflammatory reaction in previously irradiated skin, most often triggered by taxanes and anthracyclines, with occasional reports involving cisplatin, targeted therapies, and immune checkpoint inhibitors [22,23]. The incidence of chemotherapy-induced recall reactions has been estimated at 8.8%, and proposed mechanisms include hypersensitivity of irradiated stem cells or cytokine-mediated inflammation [22]. A review of 129 cases reported a median interval of eight weeks (range, 2-132 weeks) from radiotherapy to drug exposure, with RRD typically appearing five days after drug administration (range, 2-56 days) [24]. More than half of the cases in the review involved breast cancer, most frequently affecting the breast and chest wall (47%). In single-drug RRD, the median prior radiation dose was 45.0 Gy (range, 30.0-63.2 Gy) [24], though a threshold as low as 20 Gy has been proposed [23]. In our patient, cisplatin was administered at cumulative radiation doses of 2 Gy, 40 Gy, and 66 Gy. The posterior neck reaction became pronounced at 54 Gy, when this region had already moved outside the high-dose field. While the atypical timing makes RRD less likely, it cannot be completely excluded. The more plausible explanation is conventional radiation dermatitis intensified by autoimmune susceptibility, with RRD as a secondary consideration.

Assessing the activity of the underlying autoimmune disease is critical when considering radiotherapy in patients with CVDs. In the present patient's case, a more thorough evaluation of SjS disease activity might have been warranted. Although the patient lacked overt symptoms such as arthralgia and skin rash before undergoing chemoradiotherapy, elevated serum IgG levels were noted despite ongoing corticosteroid therapy, indicating potential underlying immunological activity. In similar cases, a comprehensive pre-treatment assessment that includes both clinical evaluations and laboratory parameters should therefore be considered.

The European Alliance of Associations for Rheumatology Sjögren's Syndrome Disease Activity Index (ESSDAI) is a validated tool used to assess disease activity in SjS, covering 12 domains across various organ systems. The total score ranges from 0 to 123 points, with ≤4 points indicating low disease activity, 5-13 points indicating moderate activity, and ≥14 points indicating high activity [25,26]. Incorporating the ESSDAI into the pre-radiotherapy evaluation could help tailor treatment plans for patients with SjS, potentially reducing treatment-related risks. In this case, we attempted to retrospectively calculate the ESSDAI score; however, it could not be determined because not all items relevant to the score had been accurately assessed at the time chemoradiotherapy was initiated at our institution. In our patient, the markedly elevated IgG level is consistent with persistent B-cell activation, a hallmark of SjS, and is known to correlate with higher disease activity scores within the immunological domain of the ESSDAI. The presence of increased IgG may therefore reflect ongoing autoimmune activity contributing to the patient’s heightened susceptibility to treatment-related toxicities. By contrast, C-reactive protein (CRP) is not incorporated into the ESSDAI scoring system and is not considered a direct indicator of disease activity in SjS. Nevertheless, the observed CRP elevation may still represent nonspecific inflammatory processes that cannot be fully explained by SjS alone. Alternatively, the CRP elevation could be partly attributable to other coexisting conditions; however, no infectious source was identified, and the patient did not report arthralgia at the time of presentation. While these possibilities cannot be entirely excluded, the observed CRP increase was more likely driven by tumor-associated inflammatory activity, particularly given the rapid progression of the lesion at the initiation of treatment, suggesting that the systemic inflammatory status in this patient was predominantly influenced by the malignancy, with potential multifactorial contributions.

Although surgery is also a standard treatment option for HPV-positive oropharyngeal carcinoma, in this case, definitive chemoradiotherapy was selected because surgical resection would have required extensive pharyngeal wall reconstruction with a prolonged waiting period, and the tumor was rapidly progressing. Given the favorable prognosis of HPV-positive oropharyngeal cancer treated with chemoradiotherapy, consideration could be given to reducing the cisplatin dose intensity or the total radiation dose, particularly in patients at higher risk of toxicity [27]. Significant acute toxicities arose in our patient's case, suggesting that dose reduction could have been contemplated during treatment. Nevertheless, less intensive strategies, such as unilateral neck irradiation or radiotherapy alone, might only be considered in carefully selected Stage I HPV-positive oropharyngeal carcinoma patients. In our patient, adverse features including extranodal extension, internal necrosis, and a risk of contralateral nodal involvement were present despite clinical staging as N1. Therefore, standard-dose chemoradiotherapy with bilateral neck irradiation was selected to maximize locoregional control, even at the expense of increased toxicity risk. Moreover, the use of IMRT might help reduce treatment-related toxicities [28]. However, at our institution at that time, limited manpower prevented immediate initiation of IMRT, and rapid tumor progression necessitated a prompt start of treatment with 3D-CRT. Although a switch to IMRT was considered mid-course due to escalating toxicities, the patient's deteriorating general condition precluded re-simulation and re-planning.

Limitations

In this case, the possibility of coexisting connective tissue diseases other than SjS was not thoroughly evaluated at the initial presentation because the patient demonstrated typical symptoms and test results consistent with SjS. Similarly, at the time of chemoradiotherapy at our institution, there were no evident joint deformities, skin rashes, renal involvement, cytopenias, or other systemic manifestations. Clinical signs of systemic sclerosis, such as skin thickening, digital ulcers, or interstitial lung disease, were also absent. However, comprehensive autoantibody testing was not performed, so the coexistence of other connective tissue diseases cannot be definitively excluded. This concern is supported by epidemiological data; Patel et al. reported that SjS accompanies SLE in 6.5-19%, RA in 4-31%, and SSc in 14-20.5%, all categorized as secondary SjS [29]. These observations underscore the importance of carefully considering potential overlap syndromes, even in cases that initially appear consistent with primary SjS.

## Conclusions

This case demonstrates that patients with SjS undergoing definitive chemoradiotherapy for head and neck cancer may experience severe treatment-related toxicities. While increased radiation-related risks have been seen in conditions such as SLE, SSc, and rheumatoid arthritis, the risk profile in SjS remains less clear. It is essential to further accumulate clinical data in order to clarify the safety and toxicity profile of radiotherapy in patients with CVDs and to inform individualized treatment approaches. Importantly, in light of the severe toxicity observed in this case, surgery should be clearly recommended as the preferred treatment modality over definitive radiotherapy whenever feasible in future patients with SjS.
